# Histopathologic Features for Overall Survival in Merkel Cell Carcinoma: A Case Series with Intact Mismatch Repair Protein Expression

**DOI:** 10.5146/tjpath.2023.01603

**Published:** 2023-09-15

**Authors:** Selin Kestel, Betul Ogut, Mehmet Arda Inan, Ozlem Erdem

**Affiliations:** Department of Pathology, Gazi University Faculty of Medicine, Ankara, Turkey

**Keywords:** Merkel cell carcinoma, Mismatch repair protein, Overall survival, Histopathology

## Abstract

***Objective: *** In a study of Merkel cell carcinoma (MCC), a fusion transcript between *MLH1* and *SPATA4* was identified. This fusion has the potential to generate the inactive or dominant-negative form of the protein. Therefore, we aimed to investigate whether mismatch repair protein deficiency occurr in MCC cases or not, in addition to the overall survival association with histopathologic features.

***Material and Methods:*** A retrospective review of 15 patients diagnosed with a biopsy-proven Merkel Cell Carcinoma between 2012 and 2019 was performed. Mismatch repair (MMR) protein expressions were evaluated by immunohistochemistry.

***Results: *** The median follow-up time was 36 months (mean 41, range 2-103 months). Six (40%) patients died during follow-up. The overall survival (OS) at 1 year, 2 years, 3 years, and 5 years were 87%, 80%, 62%, and 53%, respectively. The patients diagnosed at <60 years had an improved OS compared to those ≥60 years of age (p=0.016). Patients in clinical stage I had better OS than patients in clinical stage IV (p=0.011). Cases with pathological tumor stage (pT) 1 had better OS than pT3 and pT4 (p=0.045). Adjuvant radiotherapy or adjuvant radiotherapy+chemotherapy treatment improved OS compared to adjuvant chemotherapy (p=0.003). MMR protein nuclear expression was intact in 12 cases available for immunohistochemical study.

***Conclusion:*** To the best of our knowledge, this is the second study that preferentially investigated the mismatch repair protein status of Merkel Cell Carcinoma. No mismatch repair protein deficiency of MCC cases was identified in the current study.

## INTRODUCTION

Merkel cell carcinoma (MCC) is rare but one of the deadliest cancers of the skin. Its incidence has increased in recent years (1). Merkel cell carcinoma has an estimated disease-associated mortality of 33% to 46% (2). In metastatic disease, the overall survival is approximately 10 months (2,3). Toker initially described it in 1972 when he reported five cases of trabecular carcinoma of the skin with a putative origin of eccrine sweat gland (4). Although MCC is rare, its incidence has been rising due to the aging population, increased sun exposure, and the use of immunosuppressive treatments (5). About 2000 people are diagnosed with MCC annually in the United States (6).

The cellular origin for Merkel cell carcinoma is still uncertain (7). The name implies immunohistochemical and structural similarities between Merkel cells and Merkel cell carcinoma. Historically Merkel cells were thought of as the origin of MCC. However, current studies suggest four new candidates for the cellular basis: Epithelial progenitor cells, fibroblast and dermal stem cells, hair follicle stem cells, and pre/pro B cells (1,7,8). Merkel cells are postmitotic, highly specialized cells located in the basal layer of the epidermis and the external part of the hair follicle (8); they have low sensitivity for oncogenic signals. Liu et al. have demonstrated that Merkel Cell polyomavirus (MCV or MCPyV) infects and proliferates within dermal fibroblasts under certain conditions (9). Both MCC and B cells express paired box 5 (PAX5), terminal deoxynucleotidyl transferase (TdT), which is typically used for hematopoietic tumors (10,11).

**Figure 1:**
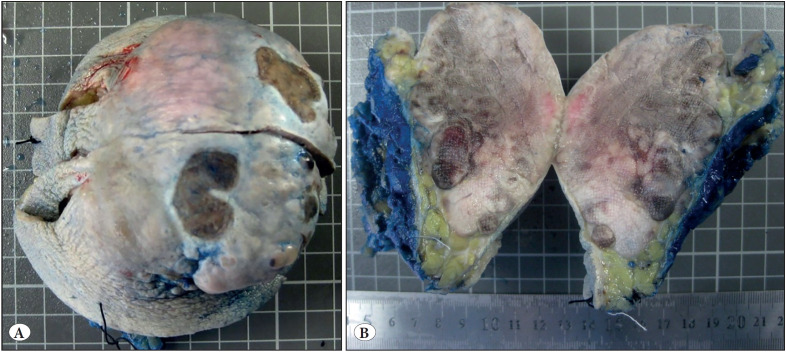
**A)** In this case, The tumor presented as a fast-growing 10 cm nodule with multifocally ulcerated, violaceous-colored overlying skin located at the right thigh. **B)** The cut surface revealed a tumor with a firm, tan brown-colored, multinodular growth pattern in the dermis and subcutis in the formalin-fixed excisional specimen.

**Figure 2:**
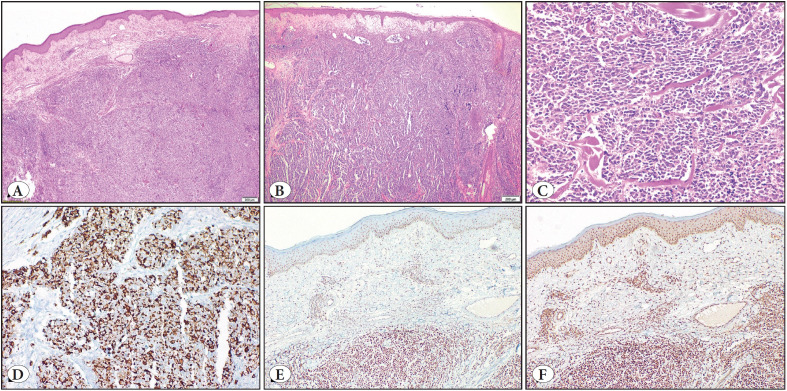
In this example **(A,B),** subepidermal edema and dermal lymphatic invasion were evident. Uniform basophilic tumor cells formed nests and diffuse sheets surrounding the adnexal structures. Dissection of collagen and some crushing artifacts accompanied tumor cells **(C)**. Paranuclear dot-like cytokeratin 20 stainings **(D).** MLH1 **(E)** and PMS2 **(F)** nuclear stainings were intact with internal controls. (Hematoxylin and Eosin stain, magnifications 40X [A, B], 200X [C], immunohistochemical stainings, magnifications 200X [CK20], 100X [MLH1, PMS2]).

There are two main pathogenic pathways for MCC development. In 2008, Chang, Moore, and colleagues found that 80% of MCC is associated with MCPyV (12). The other one, MCPyV negative, is related to UV exposure and a high mutation burden. Tumor protein p53 gene (*TP53*)*, *retinoblastoma gene (*RB*) (13), and succinate dehydrogenase D gene (*SDHD*) mutations (14) are also involved in the molecular pathogenesis of Merkel cell carcinoma. Clinically it is a rapidly growing, painless, reddish-purplish nodule (15) (Figure 1). MCC diagnosis is based on histopathological examination in most cases (Figure 2).

Traditional drug development strategies are mostly based on tumor type or a biomarker within a tumor type. In 2017, the U.S. Food and Drug Administration (FDA) approved the Anti-Programmed cell death-1 [PD-1] antibodies-Pembrolizumab as the first tissue-agnostic drug for the treatment of microsatellite instability-high or mismatch repair-deficient, unresectable or metastatic solid tumors in adult and pediatric patients (16). Although currently, tissue-agnostic drug therapies are few, patients, even in the pediatric age group with life-threatening solid tumors, may benefit from tumor-agnostic treatments selected based on the microsatellite-high/DNA mismatch repair-deficient biomarker or other few biomarkers, regardless of tumor histology or location. In a study of MCC including next-generation sequencing techniques, a fusion transcript between the mutL homolog 1 gene (*MLH1*) and spermatogenesis-associated 4 gene (*SPATA4*) was identified, and this fusion has the potential to generate the inactive or dominant-negative form of the protein (17).

In the present study, we investigated whether mismatch repair (MMR) protein deficiency also occurred in Merkel cell carcinoma cases at our center or not with immunohistochemical staining for MMR proteins (MLH1, PMS2 [postmeiotic segregation increased 2], MSH2 [mutS homolog 2], and MSH6 [mutS homolog 6]) expression. We also aimed to define known or new clinicopathological features for overall survival in Merkel cell carcinoma.

## MATERIALS and METHODS

A retrospective review of 15 patients diagnosed with a biopsy-proven MCC between 2012 and 2019 was performed (Table 1). The histopathological slides were reviewed. The following primary data were extracted: age, gender, tumor site, tumor size, lymph node status, types of treatments received, recurrence, overall survival, the primary or metastatic status of the tumor sample, ulceration, tumor thickness, lymphovascular invasion, perineural infiltration, mitotic rate, tumor-infiltrating lymphocytes (TILs), growth pattern, necrosis, adnexal infiltration, desmoplasia, nuclear chromatin pattern, and immunohistochemical studies done at the time of diagnosis (Cytokeratin 20, synaptophysin, chromogranin). When possible, additional findings, such as the patients’ medical history, were obtained from the electronic medical records or directly from the patients themselves or their relatives by a phone call. The pathological tumor stage was retroactively determined when possible using the extent of disease from the pathology reports and using the tumor size and invasion to the deep extra dermal structures according to the classification protocol developed by the American Joint Committee on Cancer, 8th edition. The correlation between clinicopathologic factors and overall survival was evaluated.

**Table 1:** Selection of the Cases

Twenty-eight tumor tissues of 15 patients were present for study with Merkel Cell Carcinoma diagnoses
↓
Clinicopathologic information of 15 patients’ from pathology reports was recorded for statistical analyses
↓
Slide recruitment from the archive for histopathologic evaluation: • Primary tumor slides were available for 13 patients. • Only metastatic tissue slides were available for one patient. • Only reactive lymph node slides were available for another patient.
↓
Paraffin-embedded tumor tissue blocks were available for 12 patients. MLH1, PMS2, MSH2, MSH6 immunohistochemical stainings were performed for 12 cases with internal and external controls.

Additionally, formalin-fixed paraffin-embedded (FFPE) tissue blocks of twelve MCC cases were retrieved from the pathology archives of our institution. Immunohistochemical expression of MMR proteins was examined using the ultraview Universal DAB detection kit on a Ventana Benchmark automated staining system. The following monoclonal antibodies were tested on immunohistochemistry: Anti-MLH-1 (clone M1, Roche), anti-MSH2 (clone G219-1129, Roche), anti-MSH6 (clone 44, Roche), and anti-PMS2 (clone EPR3947, Roche). Internal controls were positive for all cases. Only nuclear staining was scored as positive. The nuclear staining threshold required for an “intact expression” result was accepted as more than 5%. Institutional research ethics board approval was obtained for the study.

### Statistical Analysis

The overall survival data was calculated as the months from diagnosis to death from any cause or to the last follow-up for surviving patients (censored). Statistical analyses were calculated using the Statistical Package for the Social Sciences (SPSS) version 23 (IBM Corp., Armonk, NY). Descriptive statistics were calculated as mean, median, and standard deviation for quantitative variables like age, tumor size, tumor thickness, mitoses per millimeter square, and months since diagnosis. Survival analysis was calculated using the Kaplan-Meier method. Differences between survival functions were analyzed by the log-rank test. The statistically significant difference between groups was determined as *p* < 0.05.

## RESULTS

**Figure 3:**
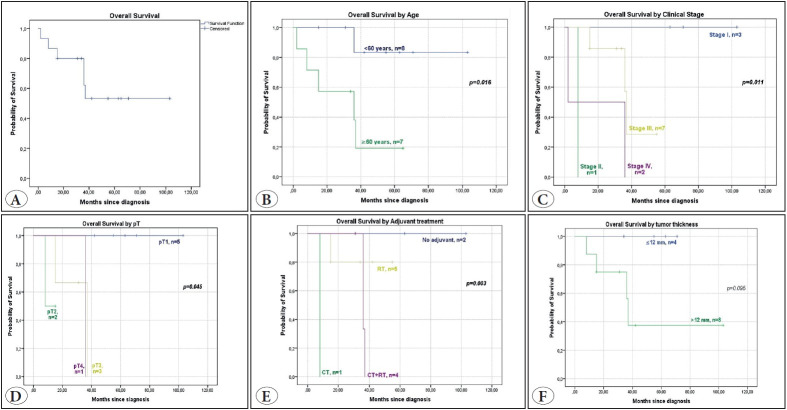
Kaplan-Meier overall survival curves for Merkel cell carcinoma patients compared for variables by age **(B)**, clinical-stage **(C),** pathological tumor stage (pT) **(D),** adjuvant treatment **(E),** tumor thickness **(F).**

There were 9 (60%) female and 6 (40%) male patients. The median follow-up time was 36 months (mean 41, range 2-103 months). Patients in this study had a mean survival of 66.3 months. Six (40%) patients were deceased during follow-up. The overall survival (OS) at 1 year, 2 years, 3 years, and 5 years were 87%, 80%, 62%, and 53%, respectively (Figure 3). The mean age at diagnosis was 65 years (median 59, range, 52-91 years). The patients diagnosed at <60 years had an improved OS compared to those ≥60 years of age (*p*=0.016) (Figure 3). Two tumors belonged to metastatic tissue, whereas 13 tumors presented with primary MCC. However, primary site and diagnosis time were known for one of the patients. Regarding their past medical history, one patient had kidney transplantation due to familial polycystic kidney disease; one patient had partial nephrectomy due to renal cell carcinoma, papillary type 2; two patients had a history of kidney stones and one of these underwent radical nephrectomy due to atrophy. In addition, two patients developed neoplasm after MCC diagnosis: one of them was ovary cancer, and the other was breast cancer. Clinical and histopathologic variables were present in Table 2 and Table 3, respectively.

**Table 2:** Univariate Analysis of Clinical Variables for Overall Survival time (months) in Merkel Cell Carcinoma, Calculated From Kaplan-Meier Analysis With Comparisons Performed With the Log-Rank Test.

	**n** **(% of total or % of total for category)**	**Exitus** **(% of total for subcategory)**	**Mean ± SE** **(95% CI) (Months)**	***P***
Overall survival	15 (100)	6 (40)	66.29±11.25 (44.24 - 88.34)	N/A
Gender	15 (100)			
Male	6 (40)	2 (33)	57.2±7.6 (42.4 - 72.0)	0.413
Female	9 (60)	4 (44)	60.3±15.6 (29.6 - 90.9)	
Age at diagnosis (year)	15 (100)			
<60	8 (53)	1 (13)	92.0±10.2 (72.0 - 112)	**0.016**
≥60	7 (47)	5 (71)	29.9±8.4 (13.3 - 46.4)	
Tumor site	14 (93)			
Head and neck	1 (7)	1 (100)	36.0±0.0 (36.0 - 36.0)	0.370
Trunk	1 (7)	1 (100)	37.0±0.0 (37.0 - 37.0)	
Extremities	12 (86)	3 (25)	79.8±11.5 (57.2 - 102.3)	
Clinical Stage	13 (87)			
Stage I	3 (23)	0 (0)	N/A a	**0.011**
Stage II	1 (8)	1(100)	N/A abc	
Stage III	7 (54)	4 (57)	N/A abd	
Stage IV	2 (15)	2 (100)	N/A b	
pT	11 (73)			
pT1	5 (46)	0 (0)	N/A a	**0.045**
pT2	2 (18)	1 (50)	N/A abc	
pT3	3 (27)	2 (67)	N/A bc	
pT4	1 (9)	1 (100)	N/A c	
Adjuvant treatment	12 (80)			
No adjuvant	2 (17)	0 (0)	N/A abc	**0.003**
Chemotherapy (CT)	1 (8)	1 (100)	N/A a	
Radiotherapy (RT)	5 (42)	1 (20)	N/A bc	
CT+RT	4 (33)	3 (75)	N/A c	
Recurrence	10 (67)			
Yes	3 (30)	2 (67)	36.5±0.5 (35.5 - 37.5)	0.271
No	7 (70)	2 (29)	78.9±14.2 (51.0 - 106.9)	

**N:** Number, **SE:** Standard error, **CI:** Confidence interval, **N/A:** Not applicable. The same letters mean there is no difference between groups

**Table 3:** Univariate Analysis of Histopathologic Variables for Overall Survival time (months) in Merkel Cell Carcinoma, Calculated From Kaplan-Meier Analysis With Comparisons Performed With the Log-Rank Test.

	**n** **(% of total or % of total for category)**	**Exitus** **(% of total for subcategory)**	**Mean ± SE** **(95% CI) (Months)**	***P***
Tumor thickness (mm)	12 (80)			N/A
≤12	4	0	N/A	0.095
>12	8	4	N/A	
Ulceration	11 (77)			
Present	2 (18)	1 (50)	36.0±0.0 (36.0 - 36.0)	0.500
Absent	9 (82)	3 (33)	72.4±14.2 (44.5-100.3)	
Mitotic rate (mm²)	14 (93)			
<10	6 (43)	1 (17)	59.5±10.5 (38.9 - 80.1)	0.106
≥10	8 (57)	5 (63)	42.6±12.6 (17.9 - 67.3)	
Lymphovascular invasion	14 (93)			
Yes	10 (72)	5 (50)	51.4±13.8 (24.3 - 78.5)	0.300
No	4(28)	1 (25)	57.0±12.1 (33.2 - 80.8)	
Perineural invasion	13 (87)			
Yes	5 (38)	2 (40)	57.2±21.9 (14.3 - 100.2)	0.396
No	8 (62)	2 (25)	57.2±8.3 (40.8 - 73.5)	
TILs	14 (93)			
Absent	2 (14)	1 (50)	36.0±0.0 (36.0 -36.0)	0.706
Present	12 (86)	5 (42)	65.1±12.8 (40.1 - 900.1)	
Growth pattern	14 (93)			
Nodular	9 (64)	3 (33)	69.8±15.2 (40.0 - 99.7)	0.560
Infiltrative	5 (36)	3 (60)	43.2±11.6 (20.5 - 66.0)	
Necrosis	14 (93)			
Extensive	2 (14)	1 (50)	25.0±12.0 (1.4 - 48.6)	0.802
Focal or absent	12 (86)	5 (42)	63.4±12.6 (38.7 - 88.2)	
Desmoplasia	14 (93)			
Present	12 (86)	5 (42)	66.9±11.9 (43.6 - 90.3)	0.180
Absent	2 (14)	1 (50)	16.5±10.3 (36.6 - 0.0)	
Adnexal involvement	12 (80)			
Present	9 (75)	4 (44)	N/A	0.122
Absent	3 (25)	0 (0)	N/A	
Nuclear chromatin	14 (93)			
Vesicular	3 (21)	1 (33)	N/A	0.326
Salt and pepper	4 (29)	3 (75)	N/A	
Hyperchromatic	7 (50)	4 (57)	N/A	

**N:** Number, **SE:** Standard error, **CI:** Confidence interval, **N/A:** Not applicable.

In terms of anatomical location, extremities (upper extremity n=6, 40%; lower extremity n=6, 40%) was the most common, followed by trunk (n=1, 7%), and face (n=1, 7%). Additionally, one metastatic MCC to the brain was present without a known primary site. One of the cases belonged to the lymph node metastatic MCC of the primary ankle MCC patient. Ulceration was present in 2 cases (18%). The mean tumor size was 57 mm (range, 18-100 mm). The mean tumor thickness was 20 mm (range, 5-60 mm). The mean number of mitoses per 1 mm² was 14 (range, 1-50). Lymphovascular invasion was present in 10 cases (72%). Perineural invasion was seen in 5 cases (38%). Tumor-infiltrating lymphocytes were present in 12 cases (86%). The growth pattern was nodular in 9 cases (64%), and infiltrative in 5 cases (36%). Necrosis was extensive in 3 (19%) cases, focal in 8 (50%) cases, and not present in 5 (31%) cases. Desmoplasia was detected in 12 cases (86%). Adnexal involvement was evident in 9 (75%) tumors. The nuclear chromatin pattern was vesicular in 3 tumors (21%), salt and pepper in 4 (29%), and hyperchromatic in 7 (50%) tumors. Lymph node metastasis was histopathologically present in 2 (25%) of 8 MCC cases. MCC cases were pathologically staged as pT1 in 5 patients, pT2 in 2, pT3 in 3, and pT4 in 1 (Figure 3). Patients were mostly diagnosed at clinical stage III. Patients in their clinical stage I had better OS than patients in clinical stage IV (Figure 3). MMR protein nuclear expression was intact in 12 cases available for immunohistochemical study. Cytokeratin 20, chromogranin, and synaptophysin stainings were characteristically positive in 14, 13, and 12 cases, respectively. The patients were treated by surgery only in 4, surgery with adjuvant radiotherapy (RT) in 5, surgery with adjuvant chemotherapy (CT) in 1, and surgery with adjuvant chemoradiotherapy (CRT) in 4 patients. Adjuvant radiotherapy or adjuvant radiotherapy+chemotherapy treatment had a better prognostic impact on OS than adjuvant chemotherapy (*p*=0.003) (Figure 3). There was no recurrence in 7 cases, whereas 3 cases recurred at 12, 16, and 20 months after the diagnosis, respectively.

## DISCUSSION

MCC is mostly seen in elderly male patients and in the head and neck regions (18). Heath et al. have reported 195 MCC cases, 168 of which were primary skin lesions. The most common location was the head and neck (n=56, 29%), followed by the lower limb (n=46, 24%), upper limb (n=40, 21%), trunk (n=16, 8%), buttock (n=9, 5%), and vulva (n=1, 0.5%) (15). In our case series, patients were mostly elderly, and extremities were the most common location. Our series included slightly more female patients, similar to another Turkish case series of MCC patients (19).

Liang et al. have reported the 2-year and 5-year OS rates as 53.9% and 32.8% in 87 MCC patients over 30 years (20). The 2-year and 5-year OS rates were 80% and 53% in our series, respectively. The two cm primary tumor size threshold did not statistically significantly affect the survival in Liang et al. and Ciążyńska et al.’s studies (20,21). In our research, less than 12 mm tumor thickness had better OS than tumors equal to or more than 12 mm (Figure 3), although this did not reach the exact level of statistical significance.

Several proofreading mechanisms are necessary to have a DNA replication system with high fidelity. A mismatch is an incorrect base pairing between an incoming deoxyribonucleoside triphosphate and the DNA template. DNA polymerase has 5’-to-3’ polymerization ability and intrinsic 3’-to-5’ exonucleolytic proofreading to prevent and correct mismatches. The third system for correcting these errors in the DNA helix from the misfit between noncomplementary base pairs is strand-directed mismatch repair. Two proteins function as heterodimers for mismatch repair in both bacteria and eukaryotes. Human MutS heterodimers (MSH2/MSH6 and MSH2/MSH3 complexes) bind specifically to a mismatched base pair. Then, human (h) MutL heterodimers (hMLH1/hPMS2, hMLH1/hPMS1, and hMLH1/hMLH3) specifically recruit proteins to remove the newly synthesized strand back through the mismatch and resynthesize DNA (22).

DNA repair is impaired when one or more mismatch repair proteins lose their function (deficient mismatch repair-dMMR). As a result, spontaneous genetic mutations accumulate in the genome leading to an increased risk of developing an increasing number of neoplasms; some are associated with familial cancer syndromes (23). Lynch syndrome is the familial cancer syndrome associated with a mono-allelic germline mutation in an *MMR* gene (24). Lynch syndrome is related to an increased risk of developing colon, rectum, endometrium, stomach, ovary, ureter, renal pelvis, brain, small bowel, and hepatobiliary tract cancers (25). Muir Torre syndrome is another familial cancer syndrome caused by mutations in the DNA mismatch repair genes with a combination of skin neoplasms (mostly sebaceous neoplasms but also keratoacanthoma) and a visceral malignancy (usually colorectal, endometrial, small intestine, and urothelial) (26). Regardless of the syndromic status, deficiency in mismatch repair proteins have been described in uterine corpus endometrial carcinoma, colon adenocarcinoma, stomach adenocarcinoma, rectal adenocarcinoma, adrenocortical carcinoma, uterine carcinosarcoma, cervical squamous cell carcinoma, endocervical adenocarcinoma, Wilms tumor, mesothelioma, esophageal carcinoma, breast carcinoma, renal clear cell carcinoma, ovarian serous cystadenocarcinoma, cholangiocarcinoma, thymoma, hepatocellular carcinoma, head and neck squamous cell carcinoma, sarcoma, cutaneous melanoma, cutaneous squamous cell carcinoma, lung squamous cell carcinoma, prostate adenocarcinoma, lung adenocarcinoma, bladder carcinoma, pediatric neuroblastoma, lower-grade glioma, chronic lymphocytic leukemia, glioblastoma, pancreatic adenocarcinoma, thyroid carcinoma, and uveal melanoma (27–33). Accompanying renal cell, breast, and ovary cancers to MCC in our case series also led to searching for the MCC and deficient MMR protein relationship.

Gambichler et al. have studied microsatellite instability in 56 MCC cases for the first time (34). Nine patients had a low level of at least one MMR protein (MLH1, PMS2, MSH2, MSH6) expression. One of them was found to have microsatellite instability-high by multiplex PCR combined with high-resolution capillary electrophoresis. They also revealed an association between low expression of mismatch repair proteins and negative MCPyV status. Nevertheless, there was no association with MMR expression and the outcome of the patients such as disease relapse or death (34). In our study, all tumors had intact expressions that were diffuse nuclear positive for MMR proteins.

Miner et al. have reported that 13 patients of cytokeratin 20-negative MCC were also negative for MCPyV by polymerase chain reaction. They also showed at least one of three cytokeratins, including cytokeratin-7, AE1/AE3, and Cam 5.2 immunoreactivity in CK20-negative MCC cases (35). Iwasaki et al. have reported additional CK20-negative MCCs. They concluded that the negativity of both cytokeratin 20 and MCPyV might be associated with poorly differentiated MCC features pertaining to their previous study that demonstrated severe nuclear atypia and pleomorphism in MCPyV-negative MCCs compared to MCPyV-positive MCCs (36,37). However, they did not find a significant relationship between CK20 negativity and MCC-specific death. Cytokeratins are intermediate-sized filament proteins found in most epithelial cells. Cytokeratin 20 (CK20) is a type I (acidic), low molecular weight cytokeratin (38). In normal Merkel cells of the skin, the CK20 arrangement is loose, leading to diffuse cytoplasmic staining (7). However, in Merkel cell carcinoma, there is a characteristic but not pathognomic CK20 staining for paranuclear collection of intermediate filaments described as paranuclear whirls, or dot-like or globoid in appearance (39,40). Some intermediate-sized filaments are immunoreactive for neurofilament in MCC (40–42). The differences in the arrangement, interaction, and regulation of intermediate filament proteins might be a candidate reason for cytokeratin-20 negativity in addition to damage of the antigenic determinants by the formaldehyde fixative.

Studies have demonstrated that MCPyV, like some other polyomaviruses, is serologically present in most adult populations. It was thought that exposure to this virus occurred during childhood (43). Furthermore, the skin microbiota also includes MCPyV, isolated from different parts of the skin surfaces by Schowalter et al. (44). MCC’s etiological relation to MCPyV and asymptomatic infection with MCPyV in healthy individuals was explained by the MCC tendency in immunocompromised subjects (12,44).

Chang et al. reported that recurrence was observed in 6 of 13 clinically node-negative and clinically followed-up MCC patients (45). Another three node-negative patients who underwent sentinel lymph node biopsy did not experience recurrence. They emphasized that wide surgical excision and initial sentinel lymph node biopsy (SLNB) are essential for improved survival outcomes in early MCC. In our study, 2 of 3 recurrent MCC cases had undergone SLNB with a positive result prior to the recurrence experience.

In the present study, the patients whose treatment history was obtained were primarily treated with surgery. When we compare adjuvant treatments, radiotherapy had an improved OS compared to chemotherapy, and RT+CT also had better OS than CT. Despite best cytotoxic chemotherapy, advanced MCC patients have 6 to 9 months of median overall survival (46). Ciążyńska et al. reported no significant impact of radiotherapy on the survival of 31 MCC patients (21). On the other hand, avelumab, an anti-programmed death-ligand-1 monoclonal antibody, was the first approved immune checkpoint inhibitor for metastatic MCC in 2017 by FDA (47). In 2018, pembrolizumab, an anti-programmed death-1 monoclonal antibody, was approved for locally advanced and metastatic MCC (47).

Ciążyńska et al. found female gender, local disease, tumor-free resection margin as independent prognostic factors for MCC (21). Since our sample is small and Cox multiple regression analysis models were insignificant, we could not test the independence of variables. However, the clinical stage was similarly significant for OS in our study on univariate analysis. All stage IV patients were deceased, and stage I patients’ prognosis was excellent, similar to our research.

The study’s main limitations were the limited number of cases and retrospective nature. The results would be more generalizable if similar studies were performed with more cases. However, the scarcity of MCC diagnoses somehow limits this process. Multi-institutional studies may provide a solution to this problem. Another limitation was the diagnosis at an advanced stage and short follow-up time due to the deceased patients. There was no information for the MCPyV status of the patients.

In conclusion, this is the second study that preferentially investigates Merkel cell carcinoma’s mismatch repair protein status to the best of our knowledge. Mismatch repair deficiency was not identified in our research. Additional prognostic findings related to OS in this study were age, clinical-stage, pathological tumor stage, and adjuvant treatment.

## Conflict of Interest

The authors declare no conflict of interest.
